# Epithelioma of the Penis

**Published:** 1907-11-23

**Authors:** 


### EPITHELIOMA OF THE PENIS.

Epithelioma of the penis is comparatively rare. Pathologically, two varieties are described: (1) a squamous-celled form which arises from the squamous epithelium of the glans penis; and (2) a columnar-celled carcinoma which derives its origin from the epithelium lining the glands of Tyson; these are situated at the corona glandis and are the source of the secretion which collects beneath the prepuce and is known as smegma. There is little doubt that, as in some other forms of malignant disease, chronic irritation predisposes to the condition, and the most important factor in producing this is the retention of the secretion behind a long prepuce. This is one of the reasons, and not the least cogent one, for performing the operation of circumcision on children with phimosis; for it is said that epithelioma of the penis is almost, if not quite, unknown among circumcised persons. In some cases warts of the penis have been known to undergo malignant change and become epitheliomatous.

Clinically, the disease may start as an ulcerating process or as an irregular papillomatous outgrowth. In either case there is eventually much tissue destruction which causes a foul blood-stained discharge to issue from beneath the prepuce. If the condition is neglected by the patient the glans penis enlarges and the growth may increase in size until the prepuce is stretched over it, or it may ulcerate through the skin. Later still the body of the penis itself is invaded, and eventually the extremity of the penis consists of a mass of sloughing malignant disease, in which it is difficult to find the situation of the external urinary meatus. The lymphatic glands are involved fairly early in the course of the disease. The inguinal glands drain the skin of the penis and are enlarged when the prepuce is attacked, but when the body of the penis is involved, secondary deposits are found in the lumbar glands.

The condition demands immediate surgical treatment by removal if the disease is recognised early, and neither inguinal nor lumbar glands are enlarged. In this case there is some hope that removal of the diseased portion will eradicate the trouble, and the penis may be amputated through the body. This is in itself not a difficult operation. A straight bougie is introduced into the urethra and the penis held vertically. A flap whose length is equal to the circumference of the penis is then marked out and reflected. This flap may be reflected from the dorsal aspect or from the skin of the under surface of the penis. The latter alternative has the advantage that urine does not tend to dribble over the surface of the wound during the process of healing. Whichever method is chosen the flap, which consists only of skin and subcutaneous tissue, is raised from the penis and its two ends joined by a straight incision round the organ. The corpora cavernosa are now cut through at the level of the base of the flap. The corpus spongiosum, however, is divided at a point about half an inch nearer the extremity. The bougie is then withdrawn. Some surgeons ligature the penis at its base with some thin indiarubber tubing before beginning the operation; but this procedure is hardly necessary because the bleeding can be easily controlled by forceps. A small opening is now made in the skin flap, and the protruding half an inch of corpus spongiosum is drawn through this. The skin flap is then fixed over the end of the penis with fine silk sutures and the orifice of the urethra sutured to the margins of the aperture in the skin flap. Before doing this it is better to slit it up vertically or horizontally or in both directions for about an eighth of an inch. This will prevent contraction during healing. The writer remembers a case in which this operation was performed for undoubted epithelioma of the penis. Some two years later the man returned with what was apparently a local recurrence of the growth on the apex of the stump, although its appearance was not typical of malignant disease. The inguinal glands were now involved, and the more radical operation, shortly to be described, was performed. When the tissue removed was examined microscopically it was found to present the appearance of a primary chancre. The case is quoted to show the danger of taking anything for granted in surgery. In this instance, the diagnosis of syphilis was not considered because it was not thought possible that coitus could have taken place.

If the disease when first seen has involved the corpora cavernosa and the lymphatic glands, it is wiser to proceed at once to more radical measures and to extirpate the entire penis. The operation is performed as follows: The patient is placed in the lithotomy position and a bougie introduced into the urethra. An incision is made in the scrotum in the middle line: this is carried right back until the urethra is exposed. The corpus spongiosum is divided about two inches in front of the triangular ligament and isolated. The incision is carried round the dorsum of the penis, and the whole organ is removed, the crura being detached from the ramus of the pubis with a periosteum elevator. The urethra is fixed in the posterior angle of the wound and the two cut edges of the scrotum are united. If the inguinal glands are enlarged they should also be removed. It is no use attempting to remove enlarged lumbar glands by means of abdominal section; since if these are so large as to be palpable through the abdominal wall the prognosis is hopeless.

If the case is first seen when the disease is in a hopelessly advanced state, and there is no chance of a radical operation being successful, the best thing to do is to amputate the end of the penis as a palliative measure. This will, at any rate, rid the patient of the fungating mass which is a source of great discomfort to him.

